# A nonsurgical treatment of peri‐implantitis using mechanic, antiseptic and anti‐inflammatory treatment: 1 year follow‐up

**DOI:** 10.1002/cre2.286

**Published:** 2020-03-17

**Authors:** Yaniv Mayer, Ofir Ginesin, Jacob Horwitz

**Affiliations:** ^1^ Department of Periodontology, School of Graduate Dentistry Rambam Health Care Campus Haifa Israel; ^2^ Periocenter Ltd. Haifa Israel; ^3^ The Ruth and Bruce Rappaport Faculty of Medicine, Technion Israel Institute of Technology Haifa Israel

**Keywords:** anti‐inflammatory, chitosan, minocycline, nonsurgical treatment, peri‐implantitis, slow release device

## Abstract

**Aims:**

The study's aim was to assess the clinical outcome 6 and 12 months after a nonsurgical treatment of peri‐implantitis per se or in conjunction with a combination of local antiseptic and anti‐inflammatory treatment.

**Materials and methods:**

Included were 69 patients with periodontitis, with 106 implants, diagnosed with peri‐implantitis. Peri‐implantitis was defined as radiographic bone loss ≥3 mm, probing depth (PD) ≥ 6 mm, with bleeding on probing. Group M peri‐implantitis was treated with ultrasonic debridement and soft tissue curettage. Group P had additional implant surface treatment with rotatory hand piece composed of chitosan bristle, soft tissue curettage combined with application of 0.95% hypochlorite and 1 mg minocycline HCl.

**Results:**

After 6 months, both groups demonstrated significant reduction of mean plaque index, PD, and clinical attachment level (0.71 ± 0.57, 0.81 ± 0.55; 4.77 ± 0.73 mm, 4.42 ± 0.5 mm; 5.03 ± 0.86 mm, 5.13 ± 0.73 mm; respectively) and bleeding on probing. After 6 and 12 months, group P showed significantly better PD results compared to group M. The bleeding was significantly less in group P after 12 months (15.3% ± 6.2, 25.1% ± 8.2, respectively).

**Conclusions:**

Adjunctive treatment with local antiseptic and anti‐inflammatories during mechanical phase was positively associated with inflammation reduction and connective tissue reattachment.

## 1 INTRODUCTION

Dental implants are valid choice for lost tooth replacement due to the high survival rate; however, biological complications are not rare. The main biological complication is peri‐implantitis, a plaque‐associated pathological condition that occurs in tissues around dental implants, which is characterized by inflammation in the peri‐implant mucosa and loss of supporting bone (Berglundh et al., 2018). Extensive bone loss might require implant explanation. The prevalence of peri‐implantitis is significant, as assessed in several meta‐analyses: Rakic et al. (2018) reported a rate of 18.5% at patient level and 12.8% at implant level (Rakic et al., 2018); Muñoz, Duque, Giraldo, and Manrique (2018) showed similar results with 17% at patient level and 11% at implant level (Muñoz et al., 2018); while Hashim, Cionca, Combescure, and Mombelli (2018) reported a wider range with 0–62.1% at implant level and 9.1–69% at patient level (Hashim et al., 2018).

Peri‐implantitis exhibits greater tissue and bone destruction compared to periodontitis (Carcuac & Berglundh, 2014; Hiyari et al., 2018), and therefore must be treated and followed more intensively. The main goals of peri‐implantitis treatment are to resolve inflammation and prevent further bone loss by decontaminating the implant surface. Treatment success is determined by no suppuration or bleeding on probing (BOP), absence of erythema and swelling, no additional bone loss, and pocket depths ≤5 mm (Berglundh et al., 2018). Treatment modalities are comprised surgical and nonsurgical procedures.

Surgical procedures range between flap surgery with or without osseous resection, to regenerative approaches using xenografts, allografts, or alloplastic materials (Keeve et al., 2019; Ramanauskaite, Becker, Juodzbalys, & Schwarz, 2018). Surgical treatments are associated with risks, adverse events, and postsurgical complications. The results of surgical treatment for peri‐implantitis are controversial in current literature (Chan, Lin, Suarez, MacEachern, & Wang, 2014; Keeve et al., 2019; Ramanauskaite et al., 2018).

Nonsurgical treatments include debridement using various devices (e.g., manual instruments, ultrasonic/sonic instruments, plastic or carbon tips, air powder, photodynamic therapy), with antimicrobial agents including systemic or local antimicrobial treatment (Estefanía‐Fresco, García‐de‐la‐Fuente, Egaña‐Fernández‐Valderrama, Bravo, & Aguirre‐Zorzano, 2019; Heitz‐Mayfield & Mombelli, 2014; Machtei, 2014; Suárez‐López Del Amo, Yu, & Wang, 2016). Outcomes of current nonsurgical treatments show limited success and low predictability (Lang, Salvi, & Sculean, 2019).

Mechanical debridement using stainless steel instruments on implant surface causes modifications of the implant surface (Keim et al., 2019; Louropoulou, Slot, & Van der Weijden, 2012), and releases titanium (Ti) particles into the surrounding tissue (Suárez‐López Del Amo, Garaicoa‐Pazmiño, Fretwurst, Castilho, & Squarize, 2018), which might cause further complications (Eger, Sterer, Liron, Kohavi, & Gabet, 2017, Fretwurst, Nelson, Tarnow, Wang, & Giannobile, 2018). This requires the use of instruments to reduce implant damage while maximizing the cleaning effect (de Tapia et al., 2019; Mann, Parmar, Walmsley, & Lea, 2012; Viganò et al., 2019). In an in‐vitro study, Keim et al. examined debridement with single device and found air powder abrasion was more efficient than sonic scaler, which in turn was more efficient than curette. Nevertheless, in all cases, unreached areas were visible (Keim et al., 2019). In the same study, air abrasion showed no surface damage, while sonic scaler and curette damaged the implant surface (Keim et al., 2019).

The aim of this retrospective study is to compare the clinical outcome of nonsurgical mechanical treatment of peri‐implantitis, as sole treatment with a combination of mechanical, and local antiseptic and anti‐inflammatory treatments, 6 and 12 months after therapy.

## 2 MATERIALS AND METHODS

### 2.1 Ethical statement

This is a retrospective, single‐center, clinical trial with a 12‐month follow‐up. The study was approved by the institutional ethical committee (0213‐19‐rmb) and conducted according to the principles outlined in the Declaration of Helsinki and Ethical Conduct for Research with Human Beings. Informed consents were obtained from all the subjects who participated in this study. The clinical trial is reported in accordance with Consolidated Standards of Reporting (CONSORT) guidelines.

### 2.2 Study population

Subjects presented at our clinic were diagnosed with periodontitis and peri‐implantitis and underwent periodontal treatment.

### 2.3 Inclusion criteria

Patients with at least one titanium implant that exhibited radiographic bone loss ≥3 mm, probing depth (PD) ≥6 mm, and BOP (Berglundh et al., 2018). Periodontal treatment, including oral hygiene instruction, followed by supra and subgingival mechanical instrumentation.

### 2.4 Exclusion criteria

No clinical documentation at 6 and/or 12 months post‐treatment; surgery was performed on the relevant sextant.

### 2.5 Treatment

**Figure 1:**
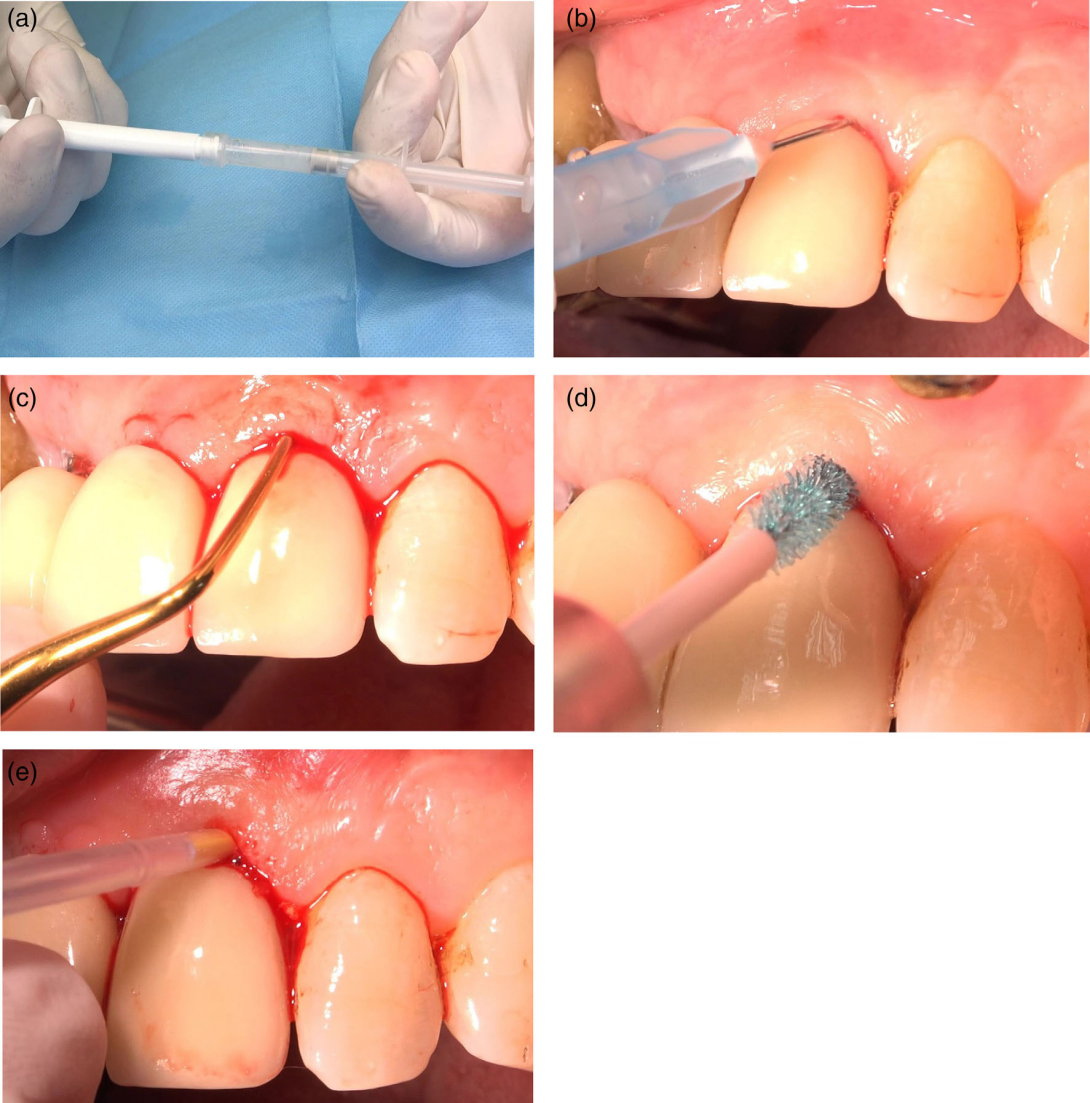
(a) Activating the solution by mixture of 0.95% sodium hypochlorite with amino acids, sodium chloride, titanium oxide, and carboxyl methylcellulose. (b) Injection of 0.95% sodium hypochlorite into the sulcus and waiting 30 s for softening the granulation tissue and prepare it for degranulation with curette. (c) Degranulation the tissue without working on the implant surface. (d) Mechanical cleaning of the implant surface with a bristle composed of a fast degrading chitosan attached to an oscillating hand piece. (e) Injection of 1 mg minocycline HCl Microspheres in to the sulcus

Periodontal treatment consisted of supra and subgingival mechanical instrumentation of the root surface with ultrasonic instrumentation after rinsing with 0.12% CHX during 1 min, under the appropriate local anesthesia. Patients were divided according to the treatment of implants with peri‐implantitis in two groups: Ultrasonic debridement with fine tips (EMS, Chemin de la Vuarpillière, 31, 1260 Nyon, Switzerland); soft tissue curettage used Teflon‐coated curettes (group M), or application of 0.95% hypochlorite with amino acids (Perisolv, RLS global AB, Mölndal, Sweden) were performed. In the group P, before use, the two components were mixed together. The sodium hypochlorite and the amino acids formed short‐lived chloramines (N‐carboxy anhydride, NCA) in a gel consistency. The gel was syringed to the pocket and filled it until overflowed. After allowing to act for 30 s, the treatment was followed by soft tissue curettage and using rotatory hand piece composed of chitosan bristle (Labrida, Oslo, Norway). The Chitosan bristle was soaked in sterile saline for at least 2 min prior to use. This made the chitosan fibers swell, and thus became soft and flexible, leading to optimal strength. The application of the hypochlorite and the curettage were repeated three times in the session. At the end, an application of 1 mg minocycline HCl (Arestin, OraPharma, NJ) (Figure 1a–e). All patients were informed before the procedure about the two therapy modalities and they had the right to decide which treatment to choose.

All patients were seen at 3‐month intervals during 1 year, as part of a routine maintenance periodontal program. Treatment outcomes were evaluated at 6 and 12 months.

### 2.6 Clinical outcomes

At baseline, 6 (T1) and 12 (T2) months, the same examiner (Y.M.) recorded the following clinical variables using a manual periodontal probe (PCP‐UNC 15; Hu‐Friedy, Chicago, IL):

- Plaque index (PI) (Silness & Loe, 1964)
- Peri‐implant (PPD), measured from the mucosal margin to the bottom of the probable pocket, and assessed at six sites per implant.
- Clinical attachment loss (CAL), measured from the implant neck to the bottom of the probable pocket, and assessed at six sites per implant.
- BOP assessed in six sites per implant.

### 2.7 Radiographic examination

**Figure 2:**
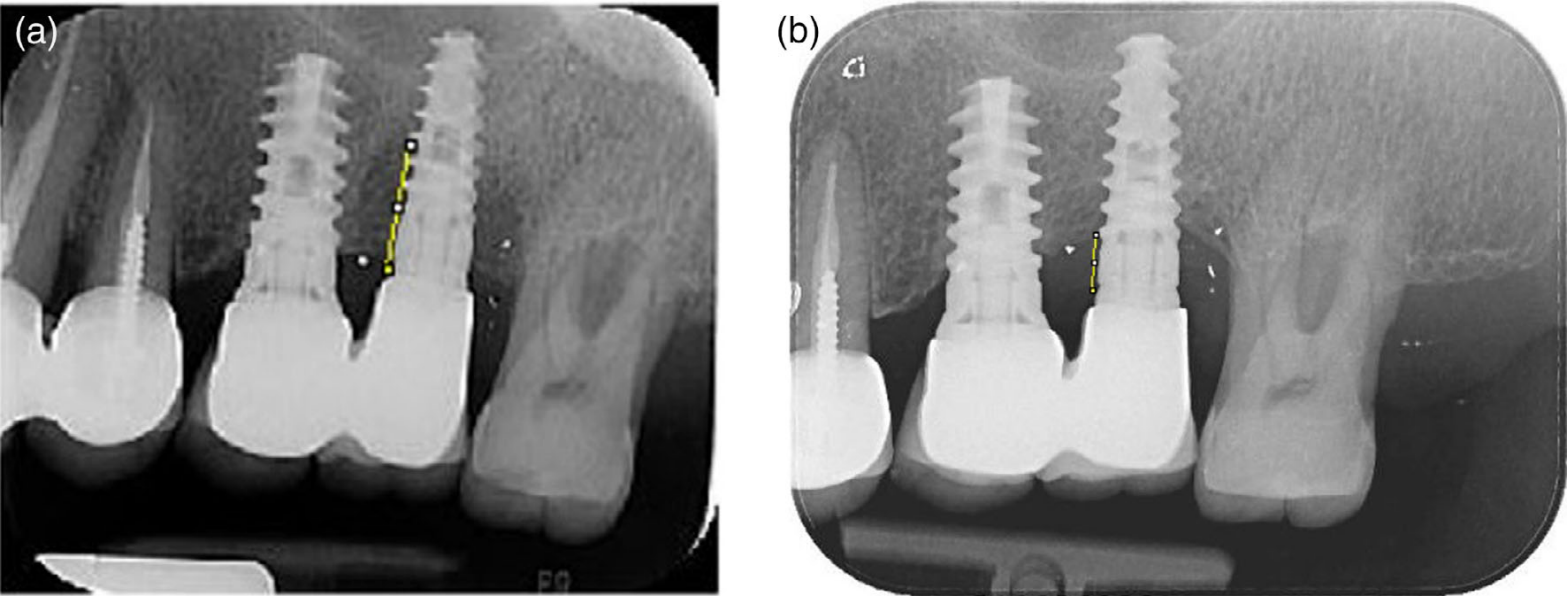
(a) Pretreatment peri‐apical radiograph. (b) 12 months' post‐treatment radiograph (group P)

- Bone level (BL) was measured from the implant‐abutment connection to the bottom of the bone defect by one examiner (O.G.), at baseline and T2, using image analysis software (ImageJ software, Java image processing program, National Institutes of Health [NIH], Bethesda) (Figure 2). In each radiograph, the length of the implant provided by the manufacturer was used to calibrate the “apico‐coronal” measurements. The distance to the coronal bone was measured at both the mesial and distal aspects of the implant.

### 2.8 Statistical analysis

Power calculation was initially performed to determine sample size. Nonsurgical therapy of peri‐implantitis can reduce pocket depth 1 mm (average). Additional reduction after using antibacterial methods reach 0.7 mm, Standard values of alpha = 0.05 and power = 80% were used. Power analysis according to these parameters yielded a sample size was of at least 32 in each group.

SPSS version 19.00 software (SPSS Inc., Chicago, IL) was used for all analyses. Primary outcome was changes in PPD at the deepest site at baseline to 6 months, and baseline to 12 months. The main outcome variable (PPD changes) and secondary variables (PI and CAL) were expressed as mean ± *SD*.

Mann–Whitney *U* test was used to compare between groups among time points (time points were not normally distributed). Level of significance was set at *p* = .05.

Independent *t* tests were used to verify differences for radiographic analysis.

## 3 RESULTS

Sixty nine patients treated during January 1, 2016–December 31, 2017 for periodontitis (grade 1–3, and stage A–B), who had a total of 106 implants with peri‐implantitis, were included. Demographic data at baseline showed no significant differences between the two groups (Table 1).

**Table 1:** Demographic data at baseline

Characteristic	M	P
Number of patients	34	35
Number of implants	52	54
Age ± *SD*	55.3 ± 6	54.2 ± 4
Male/ female	12/22	11/24
Smoker (%)	12%	10%
Implant position		
Maxilla, (%)	46%	48%
Mandible (%)	54%	52%
Type of restoration		
Screw retained (%)	34%	39%
Cemented (%)	66%	61%

*Note*: Data are presented as mean (*SD*) or percentage.

PI, PPD, and CAL at baseline, and after 6 and 12 months, are summarized in Table 2 (mean ± *SD*). PI, PPD, and CAL decreased significantly after 6 and 12 months, compared with baseline values (*p* < .001) (Table 3). No significant differences were observed after 12 months compared to 6 months for both groups.

**Table 2:** Mean clinical parameters measured at baseline, 6 months, and 12 months (mean ± *SD*)

	Baseline			6 months			12 months		
	M	P	*p* value	M	P	*p* value	M	P	*p* value
PI	1.63 ± 0.65	1.51 ± 0.63	0.36	0.71 ± 0.57	0.81 ± 0.55	0.39	0.69 ± 0.5	0.78 ± 0.5	0.38
PD (mm)	6.63 ± 1.10	6.94 ± 1.32	0.19	4.77 ± 0.73	4.42 ± 0.5	**0.006**	4.90 ± 0.66	4.57 ± 0.63	**0.01**
CAL (mm)	6.87 ± 1.18	7 ± 1.38	0.59	5.03 ± 0.86	5.13 ± 0.73	0.56	5.40 ± 0.72	5.33 ± 0.67	0.60
BOP (%)	100	100	0.6	33.2 ± 12.3	21.4 ± 14.2	0.6	25.1 ± 8.2	15.3 ± 6.2	**0.05**

*Note*: All significant results are in bold.

Abbreviations: BOP, bleeding on probing; CAL, clinical attachment loss; PI, plaque index; PPD, probing depth.

**Table 3:** Statistical significance of periodontal parameters changes among the different time points in the same groups

	Baseline–6 months		Baseline–12 months		6–12 months	
	M	P	M	P	M	P
PI	**<0.001**	**<0.001**	**<0.001**	**<0.001**	NS	NS
PPD	**<0.001**	**<0.001**	**<0.001**	**<0.001**	NS	NS
CAL	**<0.001**	**<0.001**	**<0.001**	**<0.001**	**0.002**	**<0.001**
BOP	**<0.001**	**<0.001**	**<0.001**	**<0.001**	NS	NS

*Note*: All significant results are in bold.

Abbreviations: BOP, bleeding on probing; CAL, clinical attachment loss; PI, plaque index; PPD, probing depth.

Comparison between the two treatments modality groups indicated a significant difference in PPD after 6 and 12 months (Table 4). With regard to PD, after 6 and 12 months group P showed significantly better results compared to group M alone (difference of 0.65 mm between baseline and 6 months and 0.64 mm between baseline and 12 months). No significant differences were found in CAL reduction between the two groups at the two time points. Bleeding was significantly reduced in the two groups after 6 and 12 months. Significantly, fewer sites with bleeding were found in group P during the entire follow‐up period.

**Table 4:** Differences between group P and M at two time points (Mann Whitney *U* test)

	Baseline–6 months	Baseline–12 months	6–12 months
PI	0.21	0.19	0.90
PPD	**0.02**	**0.019**	0.94
CAL	0.94	0.43	0.47
BOP	**0.001**	**0.001**	0.5

*Note*: All significant results are in bold.

Abbreviations: BOP, bleeding on probing; CAL, clinical attachment loss; PI, plaque index; PPD, probing depth.

Peri‐apical radiographs pretreatment and 12 months post‐treatment were available for limited number of implants (12 in the group P and 15 in the group M). Radiographic analysis of bone level measurements did not yield statistically significance differences between the two treatment modalities (data not shown/data on file).

## 4 DISCUSSION

This study aimed to compare clinical findings 6 to 12 months after using two nonsurgical methods of treatment for peri‐implantitis. We proposed a simple, nonsurgical treatment modality for peri‐implantitis that is easily accessible and readily available for most dental practitioners. The findings revealed a positive effect of combined protocol, including nonsurgical mechanical debridement with chitosan brushes in conjunction with local delivery of minocycline microspheres and 0.95% hypochlorite buffered with amino acids; the positive effect was maintained over the 12‐month follow‐up period. There was a synergistic effect in combining mechanical debridement as sole treatment (improved clinical parameters) with antiseptic and anti‐inflammatory treatment that further improved clinical outcome.

Biological rational of combining both materials with mechanical debridement is based on their different healing mechanisms. Hypochlorite buffered with amino acids, accompanied by mechanical debridement, disrupts the biofilm and removes granulation tissue (Roos‐Jansåker, Almhöjd, & Jansson, 2017). Minocycline HCl has an antimicrobial effect, improving probing depths and bleeding scores of pathologic peri‐implant tissue (Renvert, Lessem, Dahlén, Lindahl, & Svensson, 2006), and has a continuous effect, lasting for several days (Lee, Kweon, Cho, Kim, & Kim, 2018). Thus, initially removing granulation tissue and disrupting the biofilm increases efficiency of the antimicrobial agent. Furthermore, Minocycline HCl was proven to reduce collagenase activity, inhibit the activity of matrix metalloproteinases as well as osteoclast function, and thus prevent further periodontal destruction (Ingman et al., 1993; Vernillo, Ramamurthy, Golub, & Rifkin, 1994). Kivelä‐Rajamäki et al. (2003) showed that the antibiotic tetracycline reduced MMP‐8 (collagenase‐2) in peri‐implant sulcular fluid (Kivelä‐Rajamäki et al., 2003).

Our results are in accordance with previous studies, although higher reduction in PD and CAL were reached when comparing group M (mechanical debridement only) (Renvert et al., 2006; Renvert, Lessem, Dahlén, Renvert, & Lindahl, 2008). This difference might be due to deeper PD and CAL at baseline examination, compared to previous studies (Renvert et al., 2006, Renvert et al., 2008). The proposed combined treatment yielded greater pocket depth reduction compared to each of the treatments (2.5 mm after 6 months, 2.37 mm after 12 months). Salvi, Persson, Heitz‐Mayfield, Frei, and Lang (2007) showed improvement in PD after 6 and 12 months (1.7 and 1.7 mm, respectively) when using minocycline microspheres only (Salvi et al., 2007). Roos‐Jansåker et al. (2017) showed PD reduction of 1.75 mm after 3 months, when using hypochlorite buffered with amino acids (Roos‐Jansåker et al., 2017). Renvert et al. (2008) used minocycline microspheres in addition to mechanical debridement, compared to mechanical debridement only, and showed relative PD reduction of 0.6 mm after 12 months, supporting the use of minocycline. The results of our current study were similar, showing statistically significant differences in PD after 6 and 12 months (Renvert et al., 2008).

Systemic antibiotics are considered a valid approach to treat peri‐implantitis, in addition to mechanical debridement (Lang et al., 2019). Mombelli and Lang (1992) showed positive clinical and microbiological results after using systemic delivery of ornidazole for 10 days, with an average PD reduction of 2.55 mm after 12 months of treatment (Mombelli & Lang, 1992). Nart et al. (2019) showed similar results using Metronidazole 500 mg every 8 hr for 7 days (Nart et al., 2019), with Liñares, Pico, Blanco, and Blanco (2019) demonstrated that adjunctive administration of systemic metronidazole has shown potential effectiveness in terms of PD and radiographic defect reduction (Liñares et al., 2019). Although it might be useful, systemic antibiotic poses some risks including: superinfection (Verdugo, 2017) and antibiotic resistance (Rams, Degener, & van Winkelhoff, 2014). Proposed protocol includes local administration of antibiotics, which reduces the risk of the above mentioned complications and achieves similar clinical results compare to administration of systemic antibiotics (average pocket depth reduction of 2.37 mm in current study).

Chitosan bristle was proved to be a safe and efficient device for debridement of dental implants (Wohlfahrt, Aass, & Koldsland, 2019; Wohlfahrt et al., 2017; Zeza, Wohlfahrt, & Pilloni, 2017). Previous studies on Chitosan bristle's added value include reduced signs of inflammation (Modified Bleeding Index [mBoP] by 1.2) and probing depth (1.15 mm) (Wohlfahrt et al., 2017, Wohlfahrt et al., 2019, Zeza et al., 2017). Chitosan is an antimicrobial that relies on numerous intrinsic and extrinsic factors, such as pH, presence or absence of metal cations, pKa, molecular weight, and degree of deacetylation (Kong, Chen, Xing, & Park, 2010). In particular, Larsen et al. (2017) found that chitosan bristle significantly reduced the amount of a periopathogenic bacteria, *Porphyromonas gingivalis* (Larsen et al., 2017). Another benefit of the chitosan bristle is its ability to reach difficult to negotiate areas, due to its flexibility and long active surface. This makes superfluous any prosthetic changes (e.g., removal of prosthetic work) as most of the prosthetic work in both groups (P, M) was cemented and not screw retained (61 and 66%, respectively).

One of the causes for peri‐implantitis is residual cement, particularly in patients with history of periodontitis (Linkevicius, Puisys, Vindasiute, Linkeviciene, & Apse, 2013; Quaranta, Lim, Tang, Perrotti, & Leichter, 2017). Optional reason for the superior results of group P is cement removal, achieved in the suggested protocol in the phase of soft tissue curettage with rotatory hand piece composed of chitosan bristle. This should be further examined in future studies.

CAL did not show significant difference between the groups. This suggests that part of the improvement was due to recession of the soft tissue and part due to re‐attachment of connective tissue. Extrapolating the results suggests that 1/3 of pocket reduction was due to connective tissue reattachment and 2/3 to recession formation. This improvement is in agreement with a previous study (Roos‐Jansåker et al., 2017).

This study has limitations in terms of the relatively short follow‐up period of 12 months; longer follow‐up is required to confirm long‐term results of the treatment protocol.

Another drawback is that due to the retrospective nature of this study—availability of pretreatment and 12 months' post‐treatment radiographs were limited. This fact together with lack of personal stent might influence our ability to fully discover the radiographic changes following the suggested treatment modality. Therefore, future studies will include radiographic follow‐up.

## 5 CONCLUSIONS

Within the limitations of the present study, additional use of chitosan brush to implant surface decontamination with combined application of 0.95% hypochlorite and 1 mg minocycline HCl as part of peri‐implantitis nonsurgical treatment, resulted in statistically significant clinical improvement in terms of reduction of pocket depth after 6 and 12 months.

## 6 CLINICAL RELEVANCE

### 6.1 Scientific rationale for study

To evaluate the clinical outcome of a nonsurgical treatment of peri‐implantitis by mechanical, antiseptic, and anti‐inflammatory methods; and compare it to a mechanic treatment alone.

### 6.2 Principal findings

Both modalities showed improvement in clinical parameters after 6 and 12 months. Group P demonstrated greater reduction in pocket depth and bleeding.

### 6.3 Practical implications

Using antiseptic and anti‐inflammatory treatment during the cause related therapy at sites with peri‐implantitis can be an alternative for surgery in mild to moderate cases.

## CONFLICT OF INTEREST

The authors, therefore, declare no conflict of interests related to the content of this manuscript.

## References

[cre2286-bib-0001] Berglundh, T. , Armitage, G. , Araujo, M. G. , Avila‐Ortiz, G. , Blanco, J. , Camargo, P. M. , … Zitzmann, N. (2018). Peri‐implant diseases and conditions: Consensus report of workgroup 4 of the 2017 world workshop on the classification of periodontal and Peri‐implant diseases and conditions. Journal of Periodontology, 89(Suppl 1), S313–S318. 10.1002/JPER.17-0739 29926955

[cre2286-bib-0002] Carcuac, O. , & Berglundh, T. (2014). Composition of human peri‐implantitis and periodontitis lesions. Journal of Dental Research, 93(11), 1083–1088. 10.1177/0022034514551754 25261052 PMC4293768

[cre2286-bib-0003] Chan, H. L. , Lin, G. H. , Suarez, F. , MacEachern, M. , & Wang, H. L. (2014). Surgical management of peri‐implantitis: A systematic review and meta‐analysis of treatment outcomes. Journal of Periodontology, 85(8), 1027–1041. 10.1902/jop.2013.130563 24261909

[cre2286-bib-0004] de Tapia, B. , Valles, C. , Amaral, T. , Mor, C. , Herrera, D. , Sanz, M. , & Nart, J. (2019). The adjunctive effect of a titanium brush in implant surface decontamination at peri‐implantits surgical regenerative interventions: A randomized controlled clinical trial. Journal of Clinical Periodontology, 46, 586–596. 10.1111/jcpe.13095 30825341

[cre2286-bib-0005] Eger, M. , Sterer, N. , Liron, T. , Kohavi, D. , & Gabet, Y. (2017). Scaling of titanium implants entrains inflammation‐induced osteolysis. Scientific Reports, 7, 39612 10.1038/srep39612 28059080 PMC5216395

[cre2286-bib-0006] Estefanía‐Fresco, R. , García‐de‐la‐Fuente, A. M. , Egaña‐Fernández‐Valderrama, A. , Bravo, M. , & Aguirre‐Zorzano, L. A. (2019). One‐year results of a nonsurgical treatment protocol for peri‐implantitis. A retrospective case series. Clinical Oral Implants Research, 30(7), 702–712. 10.1111/clr.13456 31090974

[cre2286-bib-0007] Fretwurst, T. , Nelson, K. , Tarnow, D. P. , Wang, H. L. , & Giannobile, W. V. (2018). Is metal particle release associated with Peri‐implant bone destruction? An emerging concept. Journal of Dental Research, 97(3), 259–265. 10.1177/0022034517740560 29130804

[cre2286-bib-0008] Hashim, D. , Cionca, N. , Combescure, C. , & Mombelli, A. (2018). The diagnosis of peri‐implantitis: A systematic review on the predictive value of bleeding on probing. Clinical Oral Implants Research, 29(Suppl 16), 276–293. 10.1111/clr.13127 30328188

[cre2286-bib-0009] Heitz‐Mayfield, L. J. , & Mombelli, A. (2014). The therapy of peri‐implantitis: A systematic review. International Journal of Oral Maxillofacial Implants, 29(Suppl), 325–345. 10.11607/jomi.2014suppl.g5.3 24660207

[cre2286-bib-0010] Hiyari, S. , Wong, R. L. , Yaghsezian, A. , Naghibi, A. , Tetradis, S. , Camargo, P. M. , & Pirih, F. Q. (2018). Ligature‐induced peri‐implantitis and periodontitis in mice. Journal of Clinical Periodontology, 45(1), 89–99. 10.1111/jcpe.12817 28921659 PMC5774657

[cre2286-bib-0011] Ingman, T. , Sorsa, T. , Suomalainen, K. , Halinen, S. , Lindy, O. , Lauhio, A. , … Golub, L. M. (1993). Tetracycline inhibition and the cellular source of collagenase in gingival crevicular fluid in different periodontal diseases. A review article. Journal of Periodontology, 64(2), 82–88. 10.1902/jop.1993.64.2.82 8433257

[cre2286-bib-0012] Keeve, P. L. , Koo, K. T. , Ramanauskaite, A. , Romanos, G. , Schwarz, F. , Sculean, A. , & Khoury, F. (2019). Surgical treatment of periimplantitis with non‐augmentative techniques. Implant Dentistry, 28(2), 177–186. 10.1097/ID.0000000000000838 30475243

[cre2286-bib-0013] Keim, D. , Nickles, K. , Dannewitz, B. , Ratka, C. , Eickholz, P. , & Petsos, H. (2019). In vitro efficacy of three different implant surface decontamination methods in three different defect configurations. Clinical Oral Implants Research, 30(6), 550–558. 10.1111/clr.13441 31009116

[cre2286-bib-0014] Kivelä‐Rajamäki, M. , Maisi, P. , Srinivas, R. , Tervahartiala, T. , Teronen, O. , Husa, V. , … Sorsa, T. (2003). Levels and molecular forms of MMP‐7 (matrilysin‐1) and MMP‐8 (collagenase‐2) in diseased human peri‐implant sulcular fluid. Journal of Periodontal Research, 38(6), 583–590. 14632921 10.1034/j.1600-0765.2003.00688.x

[cre2286-bib-0015] Kong, M. , Chen, X. G. , Xing, K. , & Park, H. J. (2010). Antimicrobial properties of chitosan and mode of action: A state of the art review. International Journal of Food Microbiology, 144(1), 51–63. 10.1016/j.ijfoodmicro.2010.09.012 20951455

[cre2286-bib-0016] Lang, N. P. , Salvi, G. E. , & Sculean, A. (2019). Nonsurgical therapy for teeth and implants—When and why? Periodontology 2000, 79(1), 15–21. 10.1111/prd.12240 30887589

[cre2286-bib-0017] Larsen, O. I. , Enersen, M. , Kristoffersen, A. K. , Wennerberg, A. , Bunæs, D. F. , Lie, S. A. , & Leknes, K. N. (2017). Antimicrobial effects of three different treatment modalities on dental implant surfaces. The Journal of Oral Implantology, 43(6), 429–436. 10.1563/aaid-joi-D-16-00147 28972812

[cre2286-bib-0018] Lee, J. B. , Kweon, H. H. , Cho, H. J. , Kim, C. S. , & Kim, Y. T. (2018). Characteristics of local delivery agents for treating peri‐implantitis on dental implant surfaces: A preclinical study. The Journal of Oral Implantology, 45, 116–126. 10.1563/aaid-joi-D-17-00261 30452331

[cre2286-bib-0019] Liñares, A. , Pico, A. , Blanco, C. , & Blanco, J. (2019). Adjunctive systemic metronidazole to nonsurgical therapy of peri‐implantitis with intrabony defects: A retrospective case series study. International Journal of Oral Maxillofacial Implants, 34(5), 1237–1245. 10.11607/jomi.7343 31528867

[cre2286-bib-0020] Linkevicius, T. , Puisys, A. , Vindasiute, E. , Linkeviciene, L. , & Apse, P. (2013). Does residual cement around implant‐supported restorations cause peri‐implant disease? A retrospective case analysis. Clinical Oral Implants Research, 24(11), 1179–1184. 10.1111/j.1600-0501.2012.02570.x 22882700

[cre2286-bib-0021] Louropoulou, A. , Slot, D. E. , & Van der Weijden, F. A. (2012). Titanium surface alterations following the use of different mechanical instruments: A systematic review. Clinical Oral Implants Research, 23(6), 643–658. 10.1111/j.1600-0501.2011.02208.x 21564303

[cre2286-bib-0022] Machtei, E. E. (2014). Treatment alternatives to negotiate peri‐implantitis. Advances in Medicine, 2014, 487903–487913. 10.1155/2014/487903 26556414 PMC4590969

[cre2286-bib-0023] Mann, M. , Parmar, D. , Walmsley, A. D. , & Lea, S. C. (2012). Effect of plastic‐covered ultrasonic scalers on titanium implant surfaces. Clinical Oral Implants Research, 23(1), 76–82. 10.1111/j.1600-0501.2011.02186.x 21488970

[cre2286-bib-0024] Mombelli, A. , & Lang, N. P. (1992). Antimicrobial treatment of peri‐implant infections. Clinical Oral Implants Research, 3(4), 162–168. 1298430 10.1034/j.1600-0501.1992.030402.x

[cre2286-bib-0025] Muñoz, V. , Duque, A. , Giraldo, A. , & Manrique, R. (2018). Prevalence of peri‐implant disease according to periodontal probing depth and bleeding on probing: A systematic review and meta‐analysis. International Journal of Oral Maxillofacial Implants, 33(4), e89–e105. 10.11607/jomi.5940 30024992

[cre2286-bib-0026] Nart, J. , Pons, R. , Valles, C. , Esmatges, A. , Sanz‐Martín, I. , & Monje, A. (2019). Non‐surgical therapeutic outcomes of peri‐implantitis: 12‐month results. Clinical Oral Investigations, 24, 675–682. 10.1007/s00784-019-02943-8 31123873

[cre2286-bib-0027] Quaranta, A. , Lim, Z. W. , Tang, J. , Perrotti, V. , & Leichter, J. (2017). The impact of residual subgingival cement on biological complications around dental implants: A systematic review. Implant Dentistry, 26(3), 465–474. 10.1097/ID.0000000000000593 28437366

[cre2286-bib-0028] Rakic, M. , Galindo‐Moreno, P. , Monje, A. , Radovanovic, S. , Wang, H. L. , Cochran, D. , … Canullo, L. (2018). How frequent does peri‐implantitis occur? A systematic review and meta‐analysis. Clinical Oral Investigations, 22(4), 1805–1816. 10.1007/s00784-017-2276-y 29218422

[cre2286-bib-0029] Ramanauskaite, A. , Becker, K. , Juodzbalys, G. , & Schwarz, F. (2018). Clinical outcomes following surgical treatment of peri‐implantitis at grafted and non‐grafted implant sites: A retrospective analysis. International Journal of Implant Dentistry, 4(1), 27 10.1186/s40729-018-0135-5 30090967 PMC6082750

[cre2286-bib-0030] Rams, T. E. , Degener, J. E. , & van Winkelhoff, A. J. (2014). Antibiotic resistance in human peri‐implantitis microbiota. Clinical Oral Implants Research, 25(1), 82–90. 10.1111/clr.12160 23551701

[cre2286-bib-0031] Renvert, S. , Lessem, J. , Dahlén, G. , Lindahl, C. , & Svensson, M. (2006). Topical minocycline microspheres versus topical chlorhexidine gel as an adjunct to mechanical debridement of incipient peri‐implant infections: A randomized clinical trial. Journal of Clinical Periodontology, 33(5), 362–369. 10.1111/j.1600-051X.2006.00919.x 16634959

[cre2286-bib-0032] Renvert, S. , Lessem, J. , Dahlén, G. , Renvert, H. , & Lindahl, C. (2008). Mechanical and repeated antimicrobial therapy using a local drug delivery system in the treatment of peri‐implantitis: A randomized clinical trial. Journal of Periodontology, 79(5), 836–844. 10.1902/jop.2008.070347 18454662

[cre2286-bib-0033] Roos‐Jansåker, A. M. , Almhöjd, U. S. , & Jansson, H. (2017). Treatment of peri‐implantitis: Clinical outcome of chloramine as an adjunctive to non‐surgical therapy, a randomized clinical trial. Clinical Oral Implants Research, 28(1), 43–48. 10.1111/clr.12612 26013241

[cre2286-bib-0034] Salvi, G. E. , Persson, G. R. , Heitz‐Mayfield, L. J. , Frei, M. , & Lang, N. P. (2007). Adjunctive local antibiotic therapy in the treatment of peri‐implantitis II: Clinical and radiographic outcomes. Clinical Oral Implants Research, 18(3), 281–285. 10.1111/j.1600-0501.2007.01377.x 17355354

[cre2286-bib-0035] Silness, J. , & Loe, H. (1964). Peridontal disease in pregnancy. II. Correlation between oral hygiene and periodontal condtion. Acta Odontologica Scandinavica, 22, 121–135. 14158464 10.3109/00016356408993968

[cre2286-bib-0036] Suárez‐López Del Amo, F. , Garaicoa‐Pazmiño, C. , Fretwurst, T. , Castilho, R. M. , & Squarize, C. H. (2018). Dental implants‐associated release of titanium particles: A systematic review. Clinical Oral Implants Research, 29, 1085–1100. 10.1111/clr.13372 30280418

[cre2286-bib-0037] Suárez‐López Del Amo, F. , Yu, S. H. , & Wang, H. L. (2016). Non‐surgical therapy for peri‐implant diseases: A systematic review. Journal of Oral and Maxillofacial Research, 7(3), e13 10.5037/jomr.2016.7313 27833738 PMC5100638

[cre2286-bib-0038] Verdugo, F. (2017). Risk of superinfection in peri‐implantitis after systemic broad Spectrum antibiotics. International Journal of Periodontics and Restorative Dentistry. 10.11607/prd.2546 28854287

[cre2286-bib-0039] Vernillo, A. T. , Ramamurthy, N. S. , Golub, L. M. , & Rifkin, B. R. (1994). The nonantimicrobial properties of tetracycline for the treatment of periodontal disease. Current Opinion in Periodontology, 111–118. 8032451

[cre2286-bib-0040] Viganò, P. , Apaza Alccayhuaman, K. A. , Sakuma, S. , Amari, Y. , Bengazi, F. , & Botticelli, D. (2019). Use of TiBrush for surface decontamination at peri‐implantitis sites in dogs: Radiographic and histological outcomes. Journal of Investigative and Clinical Dentistry, 10(1), e12378 10.1111/jicd.12378 30474243

[cre2286-bib-0041] Wohlfahrt, J. C. , Aass, A. M. , & Koldsland, O. C. (2019). Treatment of peri‐implant mucositis with a chitosan brush—A pilot randomized clinical trial. International Journal of Dental Hygiene, 17(2), 170–176. 10.1111/idh.12381 30582880

[cre2286-bib-0042] Wohlfahrt, J. C. , Evensen, B. J. , Zeza, B. , Jansson, H. , Pilloni, A. , Roos‐Jansåker, A. M. , … Koldsland, O. C. (2017). A novel non‐surgical method for mild peri‐implantitis—A multicenter consecutive case series. International Journal of Implant Dentistry, 3(1), 38 10.1186/s40729-017-0098-y 28776288 PMC5543013

[cre2286-bib-0043] Zeza, B. , Wohlfahrt, C. , & Pilloni, A. (2017). Chitosan brush for professional removal of plaque in mild peri‐implantitis. Minerva Stomatol, 66(4), 163–168. 10.23736/S0026-4970.17.04040-7 28497660

